# Identification of NHXs in *Gossypium* species and the positive role of *GhNHX1* in salt tolerance

**DOI:** 10.1186/s12870-020-02345-z

**Published:** 2020-04-08

**Authors:** Lu Long, Jing-Ruo Zhao, Dan-Dan Guo, Xiao-Nan Ma, Fu-Chun Xu, Wen-Wen Yang, Wei Gao

**Affiliations:** 1 State Key Laboratory of Cotton Biology, School of Life Science, Henan University, Kaifeng, Henan, P. R. China; 2 State Key Laboratory of Crop Stress Adaptation and Improvement, Henan University, Kaifeng, Henan P. R. China

**Keywords:** Cotton, Soil salinization, Crop breeding, Membrane protein, Transporters

## Abstract

**Background:**

Plant Na^+^/H^+^ antiporters (NHXs) are membrane-localized proteins that maintain cellular Na^+^/K^+^ and pH homeostasis. Considerable evidence highlighted the critical roles of NHX family in plant development and salt response; however, NHXs in cotton are rarely studied.

**Results:**

The comprehensive and systematic comparative study of NHXs in three *Gossypium* species was performed. We identified 12, 12, and 23 putative NHX proteins from *G. arboreum*, *G. raimondii*, and *G. hirsutum*, respectively. Phylogenetic study revealed that repeated polyploidization of *Gossypium* spp. contributed to the expansion of NHX family. Gene structure analysis showed that cotton *NHXs* contain many introns, which will lead to alternative splicing and help plants to adapt to high salt concentrations in soil. The expression changes of *NHX*s indicate the possible differences in the roles of distinct *NHX*s in salt response. *GhNHX1* was proved to be located in the vacuolar system and intensively induced by salt stress in cotton. Silencing of *GhNHX1* resulted in enhanced sensitivity of cotton seedlings to high salt concentrations, which suggests that *GhNHX1* positively regulates cotton tolerance to salt stress.

**Conclusion:**

We characterized the gene structure, phylogenetic relationship, chromosomal location, and expression pattern of *NHX* genes from *G. arboreum*, *G. raimondii*, and *G. hirsutum*. Our findings indicated that the cotton *NHX* genes are regulated meticulously and differently at the transcription level with possible alternative splicing. The tolerance of plants to salt stress may rely on the expression level of a particular *NHX*, rather than the number of *NHXs* in the genome. This study could provide significant insights into the function of plant *NHX*s, as well as propose promising candidate genes for breeding salt-resistant cotton cultivars.

## Background

Soil salinization, caused by climate changes and human activities, is one of the most severe environmental challenges worldwide. The high concentration of salt in farmland soils was primarily from seawater and irrigation water containing toxic ions (Na^+^ and Cl^−^). Excess salts reduce the water uptake capacity of plants, block the absorption of nutrients, lead to excessive ROS production, disturb the cellular processes, and eventually inhibit plant growth [1]. The soil salinization poses a threat to the yield and quality of crops, which annually reduces arable farmland globally. On the other hand, as the world’s population is growing rapidly, modern agriculture faces the challenge of producing more products with less available farmland. The development of salt-resistant cultivars has been a major objective of crop improvement worldwide [2].

Plants have evolved elaborate mechanisms to adapt to salt stress, including ion efflux from cells and sequestration in the vacuole [3]. The ability to transport ions appears to be the key factor that determines plant adaptation in a high salt environment, and K^+^/Na^+^ transporters play a fundamental role in this process [4]. The Na^+^/H^+^ antiporters (NHXs) are membrane transporters that exchange Na^+^/K^+^ for H^+^. NHX proteins exist in a broad range of eukaryotic organisms, including yeasts, plants, and animals. In *Arabidopsis*, so far eight NHX members have been identified [5]. Based on their sequence and localization, AtNHXs are categorized into three distinct functional groups: type I localized in the vacuole (AtNHX1/2/3/4), type II in endosome (AtNHX5/6), and type III in the plasma membrane (AtNHX7/8) [5]. Growing evidence highlights the important role of NHXs in plant growth and development under diverse hostile environments, especially in saline soil.

AtNHX7, known as AtSOS1 (salt overly sensitive 1), is essential for plant survival in saline soils. It maintains a low level of toxic Na^+^ in the protoplast. The *sos1* mutant showed > 20 times higher sensitivity of sanity than wild type [6]. The ortholog of *AtSOS1* in other plant species showed similar functions, including *OsSOS1* in rice and *SlSOS1* in tomato [7, 8]. SOS1, together with SOS2 and SOS3, plays an important role in plant response to salt stress, which is conserved in plants [9]. When plants are subjected to salt stress, the high concentration of Na^+^ is perceived by cells that induce an instantaneous increase in endogenous Ca^2+^. SOS3 is a CBL (calcineurin B-Like) protein that activates SOS2 (CBL-interacting protein kinases) and forms SOS3-SOS2 complex in the presence of high Ca^2+^ [10]. The active SOS3-SOS2 complex further activates the downstream SOS1, and SOS1 encodes a Na^+^/H^+^ antiporter localized to the plasma membrane and is responsible for the extrusion of Na^+^ [6, 10].

Scientists have manipulated the expression of specific NHX isoforms to create new germplasm resources with elevated crop production in saline soils. AtNHX1 is a vacuolar-localized NHX protein that is important for osmotic regulation and plant development. Overexpression of AtNHX1 increases the salt tolerance of plant species [11–13]. For example, two NHXs (*HtNHX1* and *HtNHX2*) were isolated from a salt-tolerant plant *Helianthus tuberosus.* Heterologous expression of these genes in rice improved salt tolerance. *HtNHX2* improved grain yield as well as the harvest index of rice under both salt stress and nutrient deficient environment [13]. Type II NHXs are endosome-localized that provide salt tolerance by maintaining intracellular pH and ion balance [14]. A double mutant of the type II NHX proteins, *nhx5/nhx6,* significantly increased salt sensitivity of *Arabidopsis* [15].

Cotton (*Gossypium spp.*) is a globally cultivated crop with great economic value that provides fiber and oil. Cotton has higher natural resistance to salt compared with many other crops (rice, wheat, and maize); however, the productivity of cotton is seriously threatened by increasing soil salinization [16, 17]. Genetic engineering is an effective way to develop salt-resistant crops, and the study of the molecular mechanism of salt tolerance has provided candidate genes for genetic engineering in various species [18, 19]. While the research on salt tolerance in cotton is still at the stage of gene cloning, phenotype analysis, and function prediction [17, 20–22]. Therefore, it is necessary to further understand the salt resistance mechanism in cotton through the study of potential genes.

Plant NHX family plays an important role in salt resistance; however, research involving NHXs is still rare in cotton. The rapid development of biotechnology accelerated the molecular breeding process in cotton that allows us to study cotton NHXs in the genome-wide level [23–27]. Recently, *GhSOS1*, homologous to *AtSOS1*, was isolated from a salt-tolerant genotype of *G. hirsutum*. Silencing of *GhSOS1* significantly reduced cotton tolerance to salt, while the transgenic *Arabidopsis* with a heterogeneous expression of *GhSOS1* showed enhanced tolerance to salt as well increased expression of stress-related genes [28, 29]. In this study, we identified 47 putative NHXs from three different cotton varieties, including two diploid cotton varieties (A-genome species *G. arboreum* and D-genome species *G. raimondii*), and one tetraploid cotton variety (AD-genome species *G. hirsutum*). We further performed bioinformatics analysis, expression profiling, and virus-induced gene silencing (VIGS) to study the function of cotton NHXs. We proved that *GhNHX1*, which encodes vacuole-localized NHX protein, plays a positive role in cotton tolerance to salt. Therefore, *GhNHX1* could be used as a candidate gene for the study of molecular mechanism in cotton response to salt-stress and breeding of salt-resistant cotton varieties.

## Results

### Evaluation of salt resistance in three *Gossypium* spp.

**Fig. 1:**
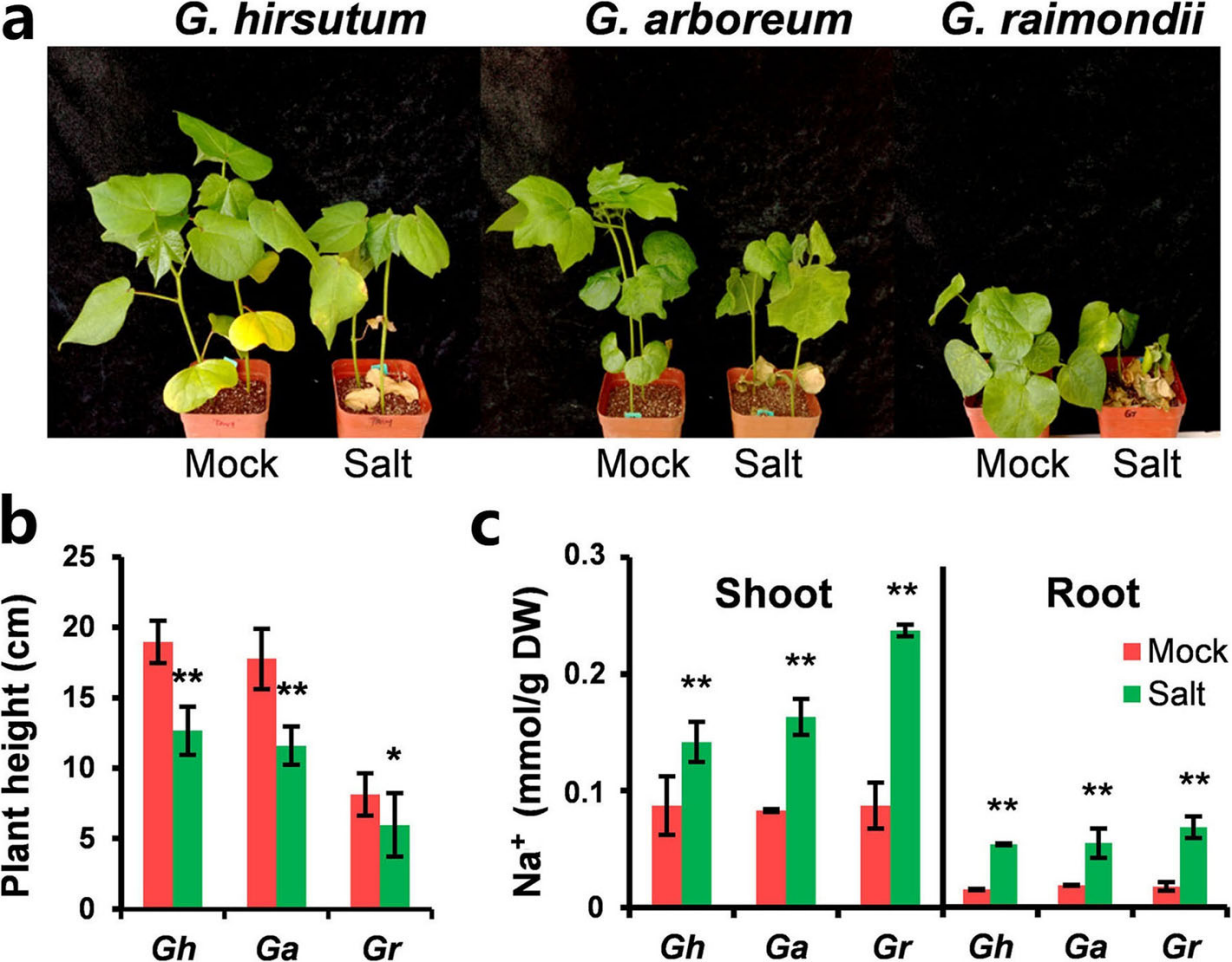
Phenotypic analysis of tetraploid and diploid cotton varieties under salt stress. **a** The symptoms on both tetraploid (*G. hirsutum*) and diploid (*G. arboreum* and *G. raimondii*) cotton varieties under salt stress. The 4-week-old cotton seedlings were irrigated with water (mock) or 300 mM NaCl solution for 12 days. **b** The plant height of mock and salt-treated cotton seedlings. **c** Quantitative measurement of Na^+^ content of mock and salt-treated cotton seedlings. The standard deviations were calculated from the results of three independent experiments (*n* ≥ 16, * *p* < 0.05, ** *p* < 0.01; *t*-test)

The seedlings of three *Gossypium* spp. (*G. arboreum* L. Shixiya1, *G. raimondii*, and *G. hirsutum* L. TM-1) were subjected to salt stress. Morphological changes such as severe dwarfing, leaf wilting, and chlorosis were observed in all three *Gossypium* spp. after irrigation with salt solution (300 mM NaCl) (Fig. 1a, b). The Na^+^ concentration in the shoot and roots of stressed seedlings was measured. Both shoot and roots accumulated high Na^+^ under salt stress (Fig. 1c). The results indicate that the seedlings of the three cotton species were all affected when grown in soils with high salt concentrations.

### Identification of cotton NHXs

NHXs are evolutionarily and functionally conserved proteins. To identify and compare the NHX members of cotton, the NHX protein sequences of *Arabidopsis*, rice, tomato, soybean, and mulberry [30–35] were downloaded from NCBI and used to build HMM to search against cotton genome database [23, 36, 37]. All putative NHXs were further analyzed by InterPro and SMART to reconfirm their signature sequences and conserved domains. The predicted protein sequences with the Na^+^/H^+^ exchanger domain were consider as cotton NHX. We identified 12, 12, and 23 NHXs from *G. arboreum*, *G. raimondii*, and *G. hirsutum*, respectively (Table 1). The details of the identified NHX proteins are listed in Table S1, including accession number, sequence length, predicted isoelectric point (*pI*), and molecular weight (Mw). The NHXs of the three *Gossypium* spp. encoded proteins that varied from 211 to 1193 AA in size with predicted *pI* ranging from 5.05 to 9.14 and Mw ranging from 22.95 to 132.84 kD.

**Table 1:** Numbers of *NHX* genes in different plant species

**Plant species**	**Scientific name**	**Total *NHX*genes identified**	**Class I Vac**	**Class II Endo**	**Class III PM**
cotton	*Gossypium hirsutum*	23	17	4	2
cotton	*Gossypium arboretum*	12	9	2	1
cotton	*Gossypium raimondii*	12	9	2	1
Arabidopsis	*Arabidopsis thaliana*	8	4	2	2
Rice	*Oryza sativa*	7	4	2	1
Mulberry	*Morus atropurpurea*	7	5	1	1
Tomato	*Solanum lycopersocum*	5	3	1	1
Soybean	*Glycine max*	11	7	3	1

### Phylogenetic tree of NHXs in cotton

**Fig. 2:**
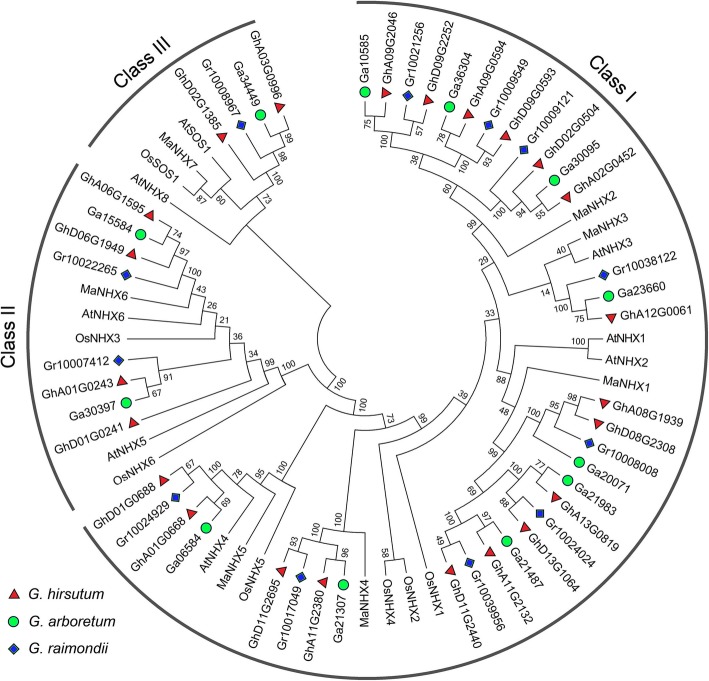
Phylogenetic relationships of diploid and tetraploid cotton NHXs. The phylogenetic tree depicts 8 NHXs from *Arabidopsis*, 7 from mulberry (*Morus atropurpurea*), 7 from rice (*Oryza sativa*), and 47 predicted NHXs from cotton. All NHXs were classified into three groups (class I–III). The unrooted tree was generated with MEGA 6.0 using the N-J method with a p-distance model (1000 replicates). Red triangle, green circle, and blue rhombus represent the *NHX* genes from *G. hirsutum*, *G. arboreum*, and *G. raimondii*, respectively. Numbers on the tree branches represent bootstrap values

To investigate the evolutionary relationships between NHXs of *G. arboreum*, *G. raimondii*, *G. hirsutum* and other plant species such as rice, mulberry, and *Arabidopsis* (Table S2), we built an unrooted phylogenetic tree using ClustalW and MEGA 6.0 with neighbor-joining (N-J) method. Similar to the previous studies in other plant species [30–34], the 47 NHX candidates of *Gossypium* were divided into three distinct classes based on the sequence conservation (Table 1, Fig. 2). The majority of NHXs (75%) of *Gossypium* spp. were categorized into class I (9 NHXs of *G. arboreum*, 9 of *G. raimondii*, and 17 of *G. hirsutum*). Class II had 2 GaNHXs, 2 GrNHXs, and 4 GhNHXs. According to previous research, class III is the smallest group, and only 1 GaNHX, 1 GrNHX, and 2 GhNHXs were categorized into this class in our study.

All NHXs of *G. arboreum* had orthologs in *G. raimondii*. For instance, Ga10585 and Gr10021256 of class I are high homologous. They shared 99.26% identity in the protein sequence with only a few single nucleotide polymorphisms observed (Figure S1) and had similar predicted *pI* and Mw values (Table S1). The phylogenetic tree and sequence alignment showed common gene duplication events associated with chromosome doubling in *G. hirsutum* compared to *G. arboreum* and *G. raimondii*, which were also found in the previous reports of gene family studies in *G. hirsutum* [38]. These gene duplication events contributed to the expansion of NHX family in *G. hirsutum*, which has twice as many NHXs in diploid cotton (Table 1). The only exception is Gr10038122 of class I. The NHX corresponding to Gr10038122 in the D sub-genome of *G. hirsutum* could be lost during the evolution process after *G. hirsutum* formation.

We further analyzed the genomic distribution of cotton *NHX*s. The genomic sequences of *NHX*s were used to query with BLAST to assess their chromosomal locations. We observed that the distribution pattern of *NHXs* in *G. hirsutum* is symmetrical and most members split evenly between A and D sub-genomes, while *NHX* genes of diploid cotton are distributed uniformly on the genome (Figure S2). The Circos cycle demonstrated the syntenic relationship between cotton and *Arabidopsis* (Figure S3), which revealed that most of the *NHX*s in *Arabidopsis* are homologous with the syntenic genes pairs in cotton. For example, *AtNHX7* located on At2 chromosome of *Arabidopsis* is highly homologous to *GhD02G1385* and *GhA03G0996* in D02 and A03 of *G. hirsutum*.

### Structural divergence of cotton *NHX* genes

**Fig. 3:**
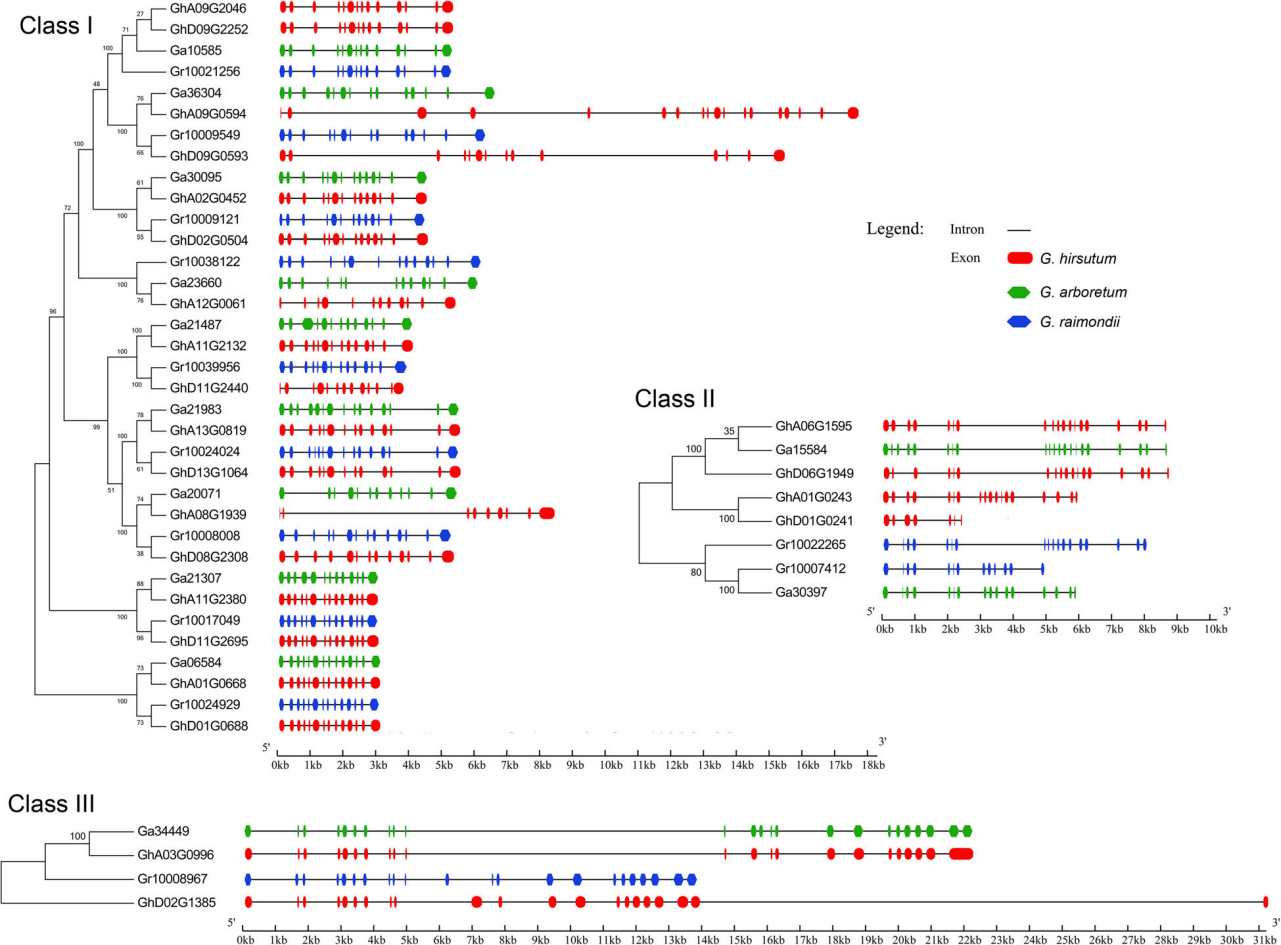
Genome organization of *NHX*s in diploid and tetraploid cotton. Phylogenetic tree of NHXs (left) and genome organization of *NHX* genes (right) of three different varieties. The phylogenetic tree was built with MEGA 6.0 using the N-J method with a p-distance model (1000 replicates). The exon-intron organization was analyzed with Gene Structure Display Server 2.0 and is proportionally displayed according to the scale; the black lines indicate introns and the boxes with different colors represent exons

The structural analysis of cotton *NHX* gene family revealed that all the identified *NHX*s possess several introns (Fig. 3). Generally, the class I *NHX* genes from *G. arboreum* and *G. raimondii* are conserved and contain 13 introns. Contrarily, one-third of the class I *GhNHXs* evolved different structure compared to the orthologous in *G. arboreum* or *G. raimondii* (eg., *Ga36304*/*GhA09G0594*/*Gr10009549*/*GhD09G0593*). *Ga36304* and *Gr10009549* with conserved protein sequence and gene structure contain 14 exons, and the longest intron is 1 kb and 0.85 kb, respectively. On the other hand, *GhA09G0594* and *GhD09G0593* evolved several extremely long introns. The longest intron of *GhA09G0594* is 3.8 kb and *GhD09G0593* is 5.2 kb. As a consequence, the genome sequences of these two *GhNHX* genes expanded to 18 kb and 16 kb, respectively, and the genome sequences of *Ga36304* and *Gr10009549* are both 7 kb. Besides, the predicted protein sequences of these four orthologs were different (Figure S4). Similar evolutionary events were observed in class III. *Gh02G1385* has a 17.2 kb intron at the 3′ end and a total genome sequence of 31 kb. These findings revealed the complexity of the gene structure of cotton *NHXs* that may be related to the biological roles of NHX family.

### Expression pattern of *NHXs* in cotton under salt stress

**Fig. 4:**
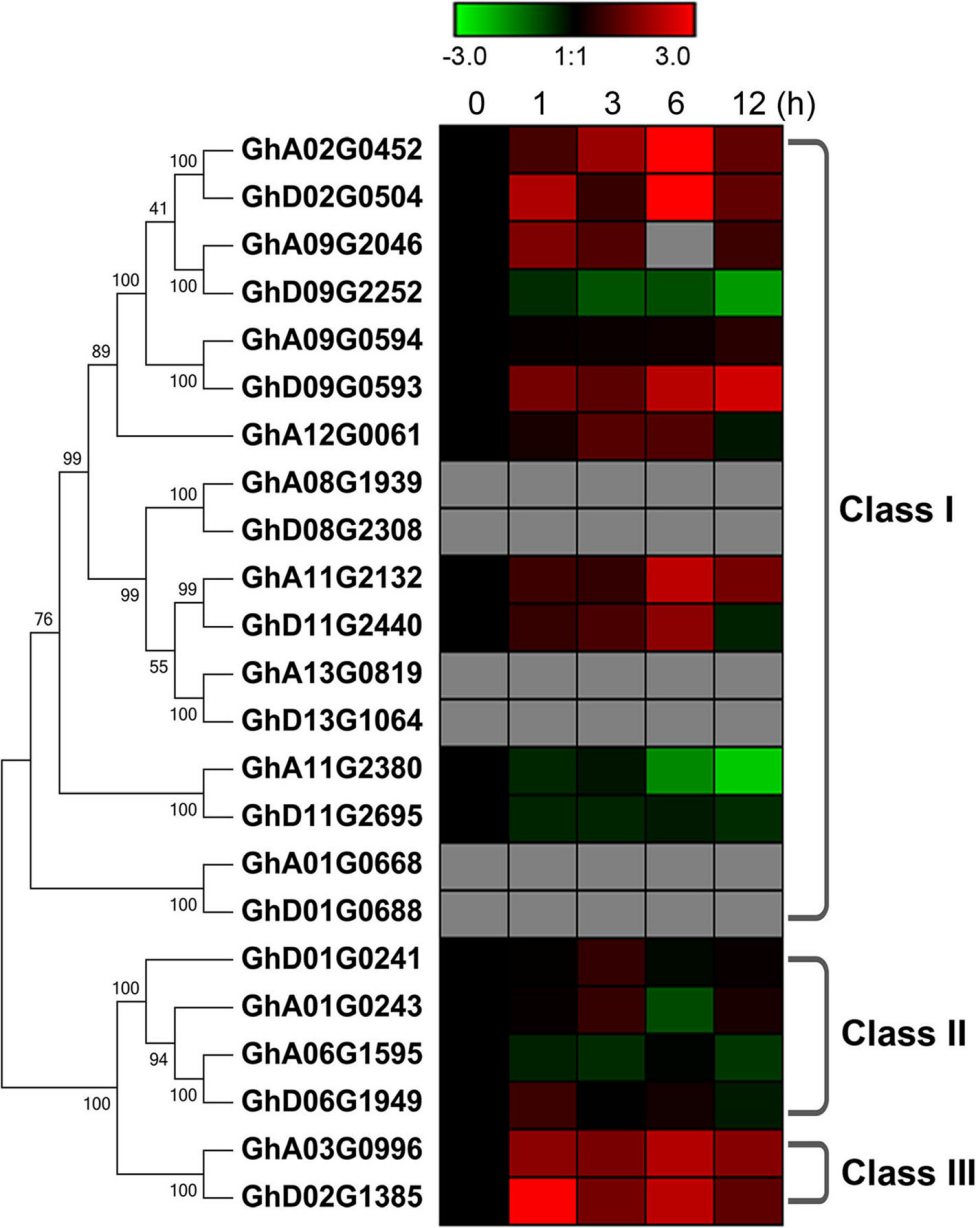
Heat map showing differential expression of *NHXs* under salt stress in *G. hirsutum*. Expression values (fold change) were log_2_ transformed, and the color scale (− 3.0, 1:1, 3.0) represents the expression values

The expression pattern of *NHX* genes in *G. hirsutum* under salt stress was studied using previously published transcriptome data [23]. RPKM (reads per kilobase per million mapped reads) values in the leaves of *G. hirsutum* under NaCl treatment at indicated time points were used to create the salt-induced heat map of *GhNHX*s. As shown in Fig. 4, The 2 genes from class III, homologous to *SOS1*, were upregulated within 1 h of salt treatment. Four genes from class II showed no significant difference. *NHX* members of class I showed significantly different expression patterns. Seven genes were significantly induced and 3 genes were significantly suppressed under salt stress, while 6 *NHX*s were not expressed in all samples. These findings indicated differences in the potential roles of NHX classes under salt response. We cloned the gene pair *GhA11G2132*/*GhD11G2440* from TM-1 transcripts for further study.

**Fig. 5:**
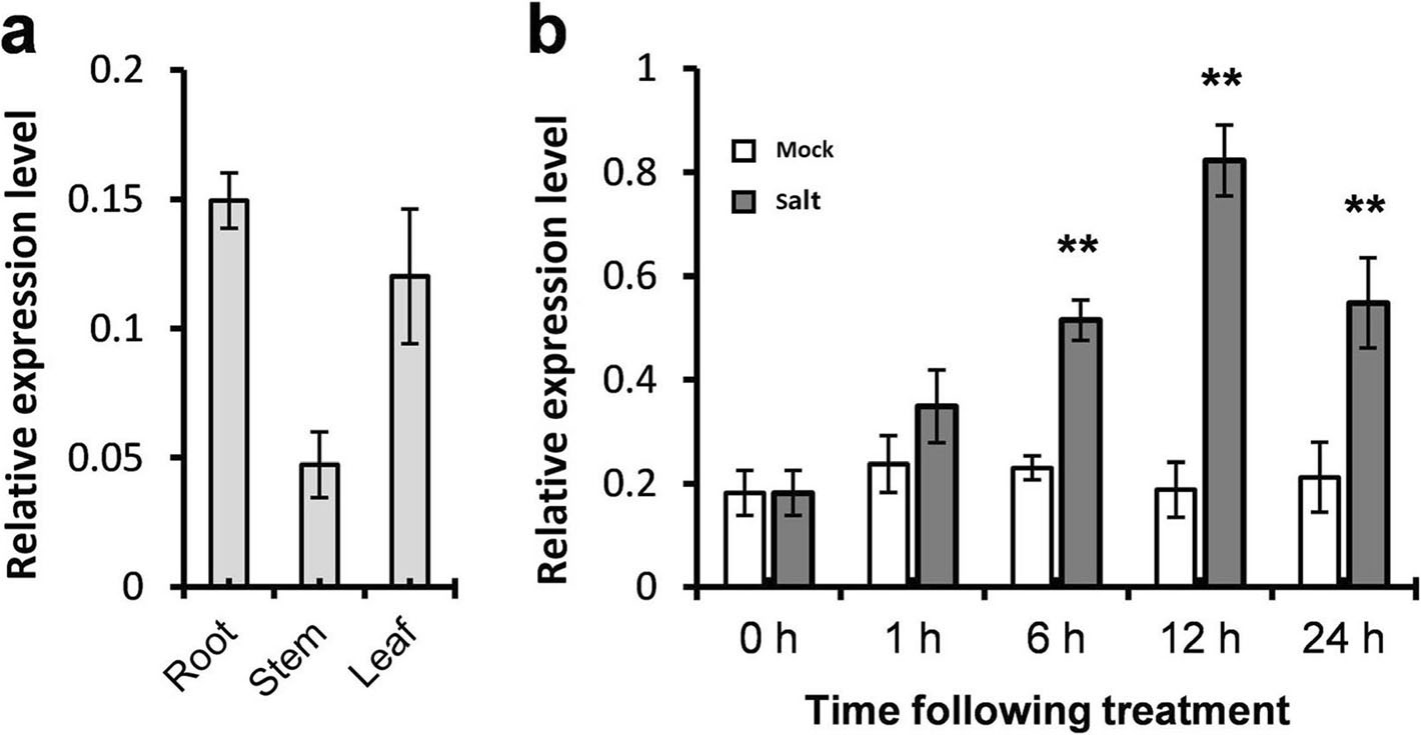
Expression profiling of *GhA11G2132*/*GhD11G2440* by qPCR analysis. **a** Expression of *GhA11G2132*/*GhD11G2440* in the vegetative organs of young cotton seedlings. **b** Expression of *GhA11G2132*/*GhD11G2440* in leaves under salt stress. *Ubiquitin 7* (*UB7*, Accession: DQ116441) was used as internal control. The standard deviations were calculated from the results of four replicates (** *p* < 0.01, *t*-test)

qPCR was performed to reconfirm the expression of *GhA11G2132*/*GhD11G2440* in different tissues and in respond to salt treatment. Because of the high similarities of *GhA11G2132*/*GhD11G2440* gene pair and high requirements for qPCR primers, it is very difficult to study the expression changes of the two genes individually with qPCR-based methods. The gene-specific primers were designed to amplify the *GhA11G2132* and *GhD11G2440* together. As shown in Fig. 5, *GhA11G2132*/*GhD11G2440* gene pair was expressed in root, stem, and leaf, and the expression level of *GhA11G2132*/*GhD11G2440* in leaf and root was two times than that in stem. *GhA11G2132*/*GhD11G2440* was significantly induced by 300 mM NaCl, which was consistent with the results of heat map analysis.

### Analysis of GhNHX1A/D protein sequence

**Fig. 6:**
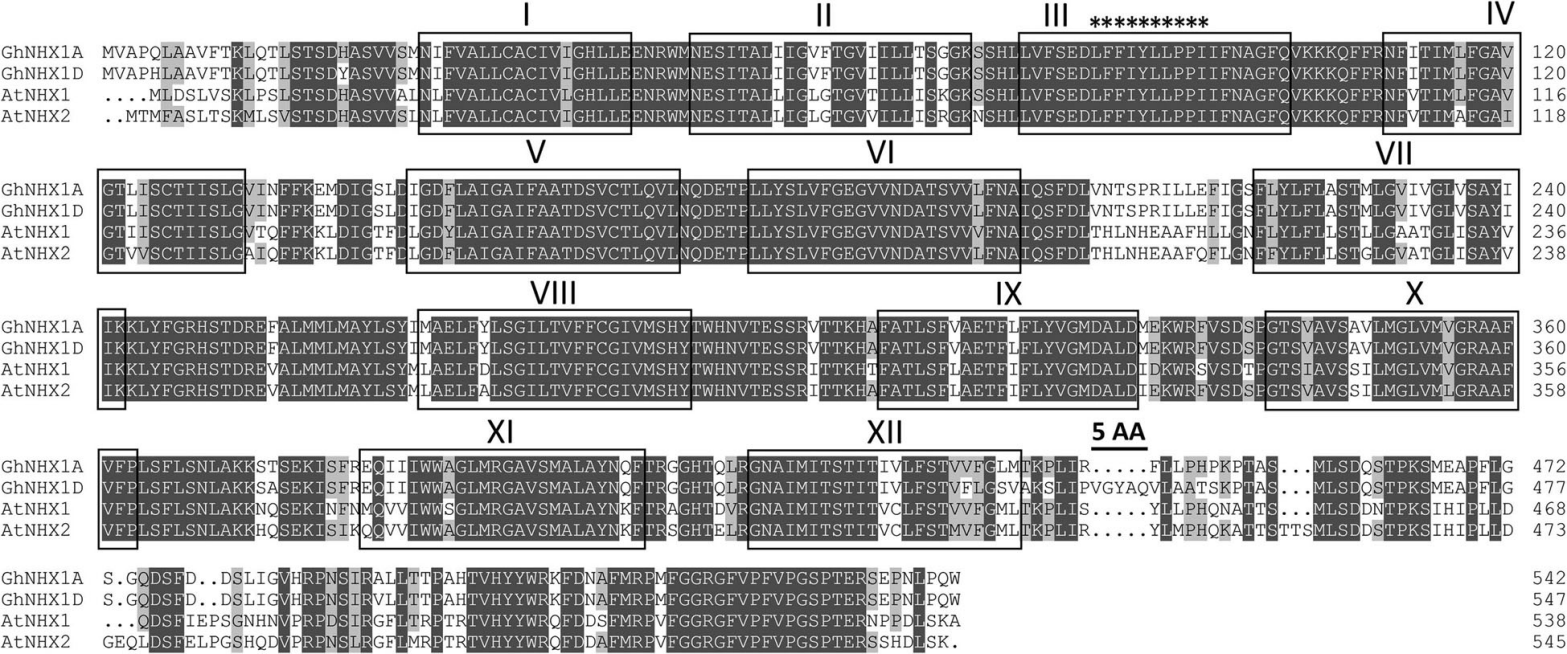
Multiple sequence alignment of GhNHX1A/D and its homologs from *Arabidopsis*. Identical amino acids are indicated with dark grey background and similar amino acids with light grey background. The predicted transmembrane domains are in shaded boxes and denoted with Roman letters I–XII. The extra five amino acids of GhNHX1D are labeled with black line and the putative amiloride-binding sites (LFFIYLLPPI) are marked with asterisks

The sequences of GhA11G2132/GhD11G2440 were studied. Phylogenetic tree (Fig. 2) and sequence alignment (Fig. 6) revealed that GhA11G2132/GhD11G2440 pair was homologous to NHX1 and NHX2 from *Arabidopsis*. GhA11G2132 from sub-genome A was denoted as GhNHX1A, and the GhD11G2440 from sub-genome D was denoted as GhNHX1D. Prediction methods highlighted 12 transmembrane domains for GhNHX1A and GhNHX1D, and they showed high similarity (96.17%) with an extra five amino acid segment at 444~448 AA in GhNHX1D. The putative amiloride-binding site (LFFIYLLPPI) that inhibits eukaryotic NHX function was found in transmembrane domain III.

**Fig. 7:**
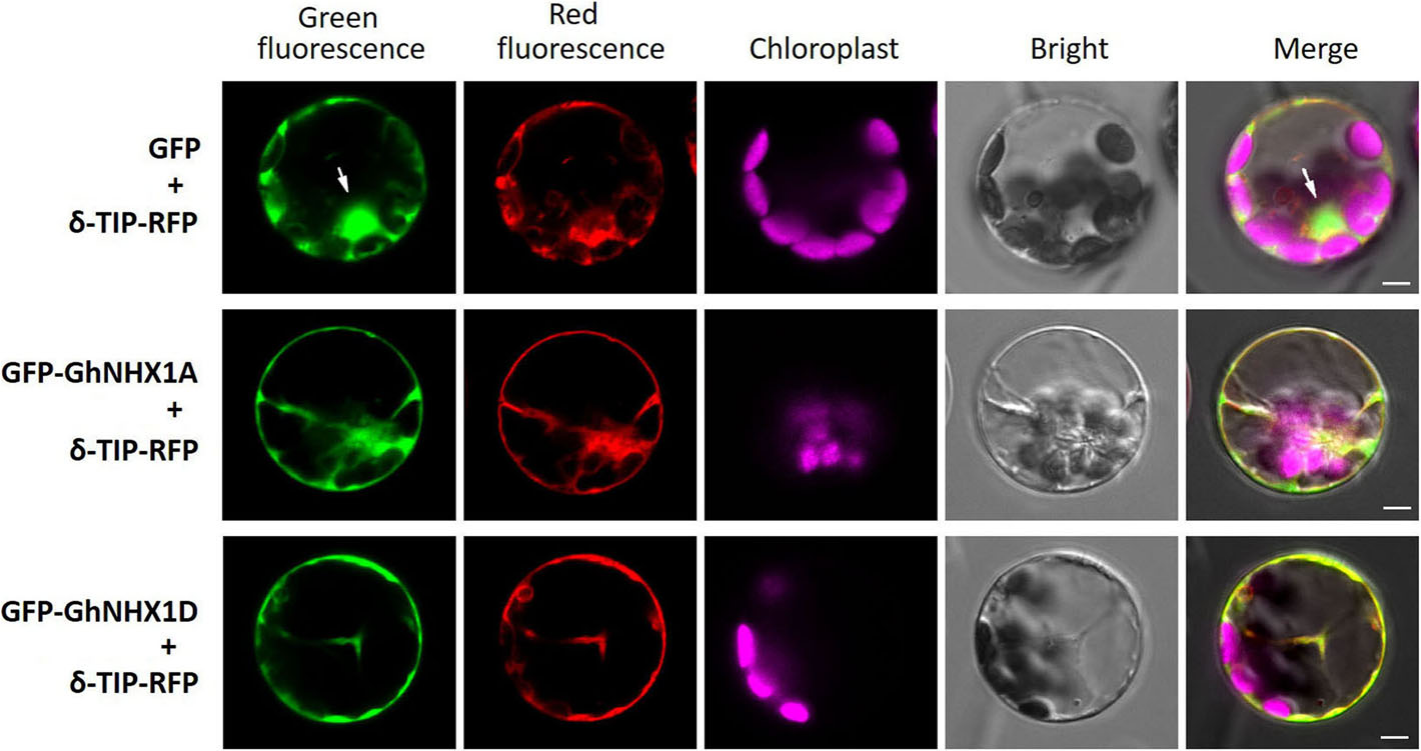
Subcellular localization of GhNHX1A/D. GFP-GhNHX1A and GFP-GhNHX1D were co-transformed with vacuolar marker δ-TIP-RFP into the *Arabidopsis* protoplasts. Images were observed with confocal laser scanning microscope. GFP coexpressed with δ-TIP-RFP was used as positive control. The cell nuclei were marked with white arrows. Scale bar = 5 μm

The biological roles of plant NHXs were closely related to their subcellular localization. To study the possible roles of the GhNHX1A/D in upland cotton, we fused GFP to the N-terminal of GhNHX1A/D. The vacuolar marker protein δ-TIP [39] fused with C-terminal RFP was cotransferred with GFP-NHX to the *Arabidopsis* protoplast. As shown in Fig. 7, GFP expressed throughout the protoplast, while red fluorescence expressed only on the vacuolar membrane in the protoplast transformed with GFP and δ-TIP-RFP vectors. The coexpression of GFP-GhNHX1A/D and δ-TIP-RFP in the protoplast, represented by overlapped red and green fluorescent signals, indicated that majority of GhNHX1A/D localized to the vacuolar system where the δ-TIP protein was located.

### GhNHX1A/D regulate salt tolerance in cotton

**Fig. 8:**
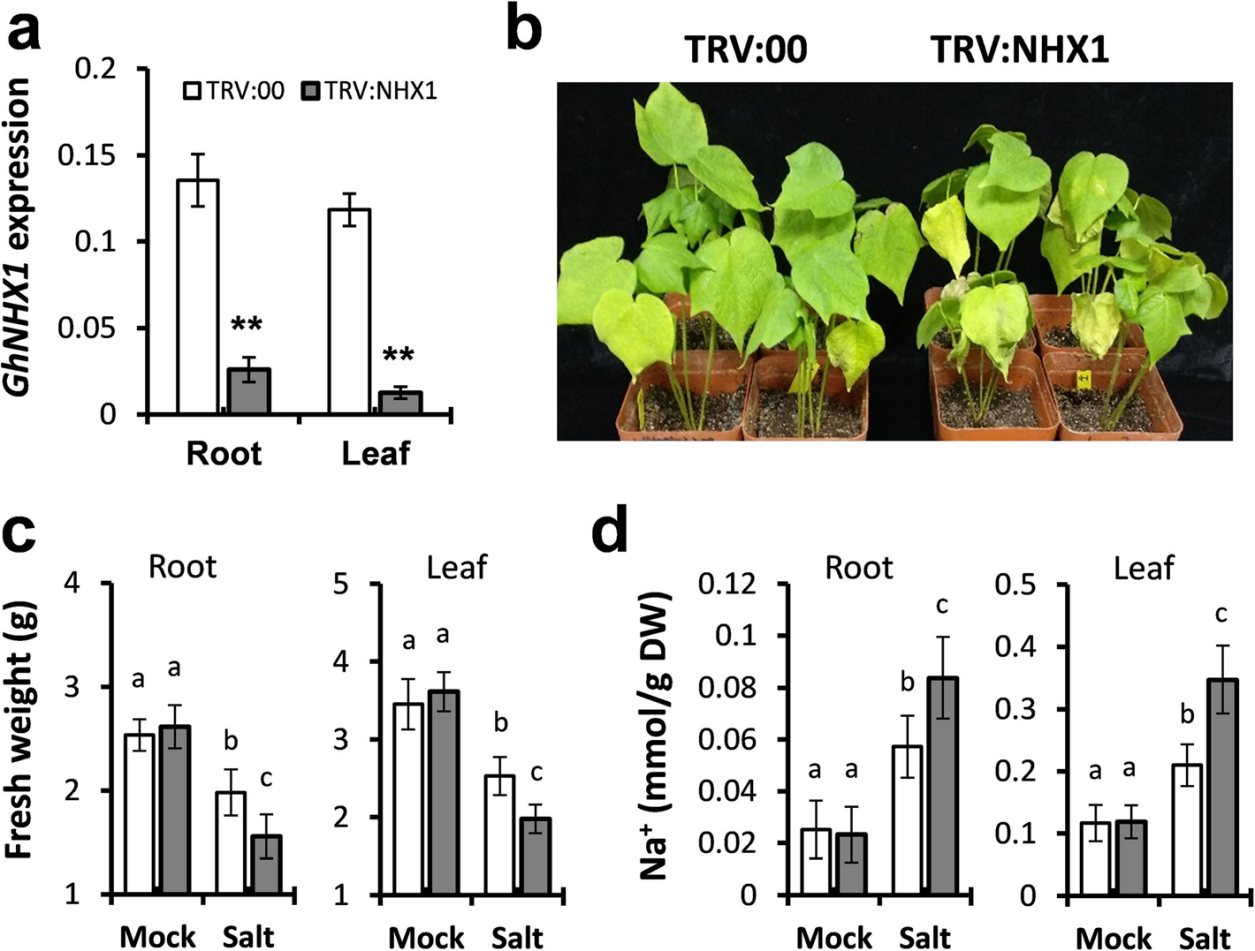
Silencing of *GhNHX1* compromises cotton tolerance to salt stress. **a** qPCR analysis of *GhNHX1* silencing efficiency in root and leaf of TRV:00 and TRV:NHX1. *UB7* was used as internal control. The standard deviations were calculated from the results of four replicates (*n* ≥ 5). **b** Representative plants of TRV:00 and TRV:NHX1 after 300 mM NaCl treatment for 6 days. **c** Quantitative measurement of biomass after water (mock) and salt treatment (*n* ≥ 14). **d** Na^+^ content of cotton seedlings after water (mock) and salt treatment (*n* ≥ 14). The standard deviations were calculated from the results of three independent experiments (*p* < 0.05)

The potential roles of GhNHX1A/D in cotton response to salt stress were studied using the VIGS method. A 376 bp fragment from 3′ end of *GhNHX1A* was cloned and inserted into the VIGS vector to silence both GhNHX1A/D. After 3 weeks, the silencing efficiency was detected in cotton leaves using qPCR. *GhNHX1* expression in *GhNHX1*-silenced (TRV:NHX1) plants reduced to 10% compared with the empty vector transferred control (TRV:00) plants (Fig. 8a). The phenotypic appearance of TRV:00 and TRV:NHX1 was studied and no significant difference was observed under normal conditions.

Cotton seedlings were subjected to water or 300 mM NaCl treatment for 6 days. High salt concentrations restricted cotton growth and development; salt treatment resulted in shorter plants with wilting and yellowing symptoms on leaves. Compared to TRV:00, the TRV:NHX1 showed reduced tolerance to salt stress with more wilting of leaves and less biomass of both roots and leaves (Fig. 8b, c). The Na^+^ content was measured in the roots and shoots of TRV:00 and TRV:NHX1 plants after water or salt treatment (Fig. 8d). After 6 days of salt treatment, the Na^+^ concentration in TRV:NHX1 was increased compared with TRV:00. These findings indicated a positive role of *GhNHX1* in cotton response to salt stress.

## Discussion

It has been reported that more than 20% of the farmland globally, including 50% of the irrigated land, is threatened by soil salinization. Most crops are sensitive to high salt concentrations that indicate the urgent need to develop new varieties with enhanced productivity in saline soils [19, 40]. Cotton is an economically important crop. Improving the yield and quality of cotton and enhancing the salt tolerance of cotton cultivars are priorities in cotton breeding [17, 41]. *NHX* genes play important roles in salt stress. Therefore, studying the function of cotton *NHXs* could provide a theoretical basis of salt tolerance mechanism and help identify the candidate genes for breeding salt-tolerant cotton.

NHX family is relatively small and conserved in plant species. In diploids, the number of *NHX* genes is around seven (Table 1) with the exception of soybean that contains 11 *NHX* genes. Soybean is a diploid crop derived from a palaeo tetraploid. This typical evolutionary process with duplications, deletions, mutations, and DNA rearrangement events resulted in 75% of soybean genes with two or more copies [42]. The diploid cotton also experienced complex polyploidy during its evolution. The repeated polyploidization of *Gossypium* resulted in a large and unexpected complex genome [36, 37, 43]. Wang et al. revealed that the ancestors of cotton experienced more multiplication events (five times) followed by extensive whole genome reshuffle and large-scale chromosome loss [44]. We identified 12 NHXs from both diploid cotton cultivars. This suggested that the number of *NHX* genes in a plant species is correlated with the size and complexity of its genome. Chromosome polyploidization also contributes to the expansion of NHX family. The heterotetraploid cotton was produced by the hybridization of A and D genomes approximately 1~2 Myr ago [23, 24, 43]. The chromosome doubling resulted in double the number of *NHX* genes in *G. hirsutum* compared to *G. arboreumii* and *G. raimondii*. The number of *NHX* genes in a plant species is not directly related to its tolerance to salt stress. There are 7 *NHXs* in both rice and mulberry; however, they exhibit different tolerance levels in saline soils. Rice is the model plant of Poaceae family that is sensitive to salt stress, while mulberry is a perennial tree that adapts to adverse environments. Compared with diploid cotton, the tetraploid cotton has twice the number of *NHX* genes; however, the tetraploid cotton showed no absolute increase in salt tolerance.

NHXs of different plant species are divided into three classes based on their sequence similarity. Previous researches highlighted the distinct functional roles and subcellular localization of plant NHXs of three classes [31, 34], which also suggest the sequence conservation of NHX orthologs among species and the close correlation between the function of NHXs and their localization. Class I NHXs localized to vacuole membrane, class II NHXs localized to the endosome, and class III NHXs localized to the plasma membrane. 75% of cotton NHXs are divided into class I, while only 50% of *Arabidopsis* members belong to class I NHXs [34]. These results revealed that the expansion of NHX family in diploid cotton was due to the large number of *NHX* genes in class I. The expression of *NHXs* in *G. hirsutum* under salt stress was studied and a distinct expression pattern was observed among three classes (Fig. 4). The 2 genes from class III, orthologous to SOS1, were induced by salt. SOS1 is pivotal for plant survival in saline soils [6], which suggests the possible role of class III *GhNHX*s in salt response. The expression of 4 *NHX*s of class II was not closely related to salt treatment. According to the previous study, the endosome-localized NHXs are involved in vesicle pH homeostasis and endomembrane trafficking [5, 14, 45]. The *GhNHX*s of class I exhibit diverse expression pattern under salt stress. These results were corroborated by the phylogenetic study, which indicates that NHXs from the three classes are regulated meticulously and differently at the transcription level.

*NHX* genes in cotton contain large number of long introns (Fig. 3), which is also found in other plant species [30–34]. The number of introns is related to the organism’s ability to adapt to adverse environmental conditions [46, 47]. The introns are molecular basis of alternative splicing. More number of introns in a gene produces more functional transcripts by alternative splicing. Alternative splicing has been considered as a powerful source of functional innovation in evolutionary adaptation. Evidence has shown alternative splicing of at least half of the human genes [48]. In fact, the studies on plant NHXs have proved high frequency of alternative splicing in post-transcriptional regulation [30]. This study provides information on the multilevel regulation of NHXs.

NHXs are closely related to environmental adaptation. The present study on NHXs has promising prospects in crop breeding for salinity. In this study, the salt-induced gene pair *GhNHX1A* and *GhNHX1D* were silenced to study their function. Both genes were upregulated after salt treatment, and their proteins were located to the vacuolar system. Silencing of *GhNHX1A* and *GhNHX1D* together resulted in reduced tolerance of cotton seedlings to high salt concentrations, suggesting that *GhNHX1* plays positive role in cotton tolerance to salt stress. These findings provide candidate genes that enable us to produce new germplasm for research and breeding of cotton cultivars resistant to salt stress.

## Conclusions

To identify salt-resistant genes and study the evolution of NHXs in cotton, bioinformatics analysis, and molecular characterization were performed. We identified 47 *NHXs* from three *Gossypium* spp.. The cotton *NHXs* contained many introns, and the complexity of gene structure may be related to the adaption of cotton to salt stress. The expression pattern of *NHX*s indicates the possible differences in the roles of distinct NHX classes under salt stress. In addition, functional analysis of *GhNHX1* using VIGS proved that *GhNHX1* is a positive factor in cotton response to salt stress. These findings could contribute to the study of cotton response to salt stress and provide candidate genes for breeding salt-resistant cotton cultivars.

## Methods

### Plant materials and salt treatment

The seeds of *Arabidopsis* Columbia (Col-0) were suspended in 0.1% (w/v) agarose solution and sown in pots filled with growth medium consisting of perlite, vermiculite, and peat. The pots were placed in a growth chamber (Conviron CMP6010, Canada) at 22/20 °C, 16 h light/8 h dark (day/night) for germination and growth.

Seeds of three different cotton varieties, including *G. hirsutum* L. TM-1, *G. arboreum* L. Shixiya1, and *G. raimondii*, were collected from the germplasm bank of the state key laboratory of cotton biology of China. The seeds were soaked in sterile water for 4 h and allowed to germinate on wet filter paper at 25 °C for 1–2 days. The germinating seeds were planted in growth medium in a growth chamber at 25/22 °C and 16 h light/8 h dark (day/night). After 4 weeks, the well-grown plants were treated with water or salt. For salt treatment, each cotton seedling was watered with 50 mL of 300 mM NaCl solution. Cotton seedlings treated with deionized water were used as mock. For phenotypic observation, photographs were taken after 12 days of treatment. Salt ion concentration was detected after photographing.

### Identification of cotton NHXs

Sequences of the NHX proteins of various plant species were downloaded from NCBI (http://www.ncbi.nlm.nih.gov/). These proteins were used as reference proteins to build a Hidden Markov Model (HMM). HMM was used as a query to search against protein database of *G. hirsutum*, *G. arboreum*, and *G. raimondii*. The nucleotide and protein sequences of *G. hirsutum*, *G. arboreum*, and *G. raimondii* were obtained from CottonGen database (https://www.cottongen.org/) [49]. The conserved domains in each candidate sequence were scanned by motif scan using SMART (http://smart.embl-heidelberg.de/smart/) and InterPro (http://www.ebi.ac.uk/interpro/). The molecular weight (Mw) and isoelectric point (*pI*) of candidate proteins were predicted using Compute pI/Mw tool (http://web.expasy.org/compute_pi/). The unrooted phylogenetic tree was built with the aligned NHX protein sequences using the neighbor-joining method in MEGA6 with 1000 bootstrap replicates with a p-distance model and partial deletion.

### Gene structure analysis

The coding sequence and corresponding genomic DNA sequence were downloaded and submitted to Gene Structure Display Server 2.0 (http://gsds.cbi.pku.edu.cn/), a web-based bioinformatics tool, to visualize gene features concerning intron-exon organization of cotton *NHX* genes.

### Genomic distribution of *NHX* genes

The chromosomal location and syntenic diagram were generated as previously reported [38]. Cotton *NHX* genes were mapped onto corresponding chromosomes by MapInspect program (http://www.plantbreeding.wur.nl/UK/software_mapinspect.html) to draw the chromosomal locations of each *NHX* genes. The syntenic analysis showing the genomic distribution of *NHX*s and the synteny relationships between cotton *NHX*s and *Arabidopsis NHX*s was performed with Circos software [50].

### RNA isolation

Total RNA was isolated from *G. hirsutum* using the EASYspin Plus Plant RNA Kit (AidLab, Beijing, China) according to the manufacturer’s instructions. Approximately 1 μg of total RNA was used for the first-strand cDNA synthesis using M-MLV Reverse Transcript System (Promega, USA). The cDNA samples were diluted 50 times and stored at − 20 °C for further experiments.

### Heat map and qPCR analysis

We obtained the released transcriptome data of *G. hirsutum* L. TM-1 from NCBI and translated the raw data into RPKM values, which represented the expression levels of *NHX*s. Heat map was generated using Genesis 1.8.1 program [51]. Four replicates of qPCR analysis were performed on an ABI 7500Fast Real-Time PCR System (Applied Biosystems, USA) with SYBR Green Master Mix (Vazyme Biotech, China) as described in our previous publication [52]. The relative fold-changes of target genes were calculated using the 2^-∆Ct^ method, with cotton *ubiquitin* 7 gene (*UB7*), amplified as an internal control to normalize the cDNA amplification in each reaction.

### Gene clone and vector construction

The target *NHX* genes were amplified by PCR and ligated to the expression vector pK7FWG2 with GFP fused to the N-terminus. The marker protein δ-TIP was fused to red fluorescence protein (RFP) [39]. The gene silencing vectors used for VIGS were constructed as described previously and was transferred into *Agrobacterium tumefaciens* [53]. The primers used in this study were designed with Primer Premier 5 software and were listed in Table S3.

### Transient transformation of *Arabidopsis* protoplast

Young leaves of *Arabidopsis* seedlings were collected and used for protoplast isolation as described previously [54, 55]. For transient transformation, 15 μg of plasmid was gently mixed with 100 μL of the protoplast (10^5^/mL) and incubated at room temperature for 30 min. Green and red fluorescence were observed using a confocal laser scanning microscope (BioRad Radiance 2100, USA).

### VIGS in cotton

Transformed *A. tumefaciens* cells containing pTRV1, pTRV2, and pTRV-NHX1 were collected and resuspended in suspension buffer. pTRV1 was mixed with pTRV2 and pTRV-NHX1 in equal amounts, and the concentration of the bacterial suspension was adjusted to 0.6–0.8 (OD_600_) and incubated at room temperature for 3 h. The *A. tumefaciens* suspension was infiltrated into the cotyledons of 1-week-old cotton seedlings by syringe infiltration. The infiltrated seedlings were kept in dark for 12 h and moved into growth incubator [56]. After 3 weeks, the cotton seedlings were subjected to gene silencing efficiency analysis and salt treatment. To study the silencing efficiency of *NHX1*, the roots and leaves of TRV:00 and TRV:NHX1 were harvested for RNA extraction and qPCR analysis. To study the salt tolerance of TRV:00 and TRV:NHX1, the TRV:00 and TRV:NHX1 seedlings were subject to water or 300 mM NaCl treatment for 6 days, the quantitative measurement of plant biomass and Na^+^ content were conducted after the water or NaCl treated TRV:00 and TRV:NHX1 seedlings being photographed.

### Measurement of Na^+^ concentration

The roots and leaves of cotton seedlings were harvested, dried, and ground into powder. Approximately, 0.05 g of the tissue powder was dissolved in 5 mL of concentrated nitric acid, and the nitric acid solution was diluted to 5% with water. The supernatant was used to detect the ion concentration using an atomic absorption spectrophotometer [57].

## Supplementary information

**Additional file 1: Table S1.** Summary of NHXs of *G. hirsutum*, *G. arboreum*, and *G. raimondii*.

**Additional file 2: Table S2.** NHX proteins used for the phylogenetic analysis.

**Additional file 3: Table S3.** The gene primers used in this study.

**Additional file 4: Figure S1.** Sequence alignment of Ga10585 and Gr10021256. The alignments were performed using DNAMAN software. Identical amino acids are in dark blue background. The sequence differences are indicated by red asterisk.

**Additional file 5: Figure S2.** Chromosomal distribution of *NHX*s from *G. hirsutum*, *G. arboreum*, and *G. raimondii*.

**Additional file 6: Figure S3.** Genome-wide synteny analysis of *NHX*s from *G. hirsutum* and *Arabidopsis*. The approximate positions of *NHX*s in chromosomes are indicated by a short gray line on the Circos circle with different colors representing different chromosomes.

**Additional file 7: Figure S4.** Sequence alignment of Ga36304, GhA09G0594, Gr10009549, and GhD09G0593. The alignments were performed using DNAMAN software. Identical amino acids are in dark blue background.

## Data Availability

The datasets used and/or analysed during the current study available from the corresponding author on reasonable request.

## Abbreviations

NHXs: Na^+^/H^+^ antiporters
ROS: Reactive oxygen species
VIGS: Virus-induced gene silencing
*pI*: Isoelectric point
Mw: Molecular weight
*Gh*: *Gossypium hirsutum*
*Ga*: *Gossypium arboreum*
*Gr*: *Gossypium raimondii*
HMM: Hidden Markov Model

## References

[1] Petrov V, Hille J, Mueller-Roeber B, Gechev TS (2015). ROS-mediated abiotic stress-induced programmed cell death in plants. Front Plant Sci.

[2] Schroeder JI, Delhaize E, Frommer WB, Guerinot ML, Harrison MJ, Herrera-Estrella L, Horie T, Kochian LV, Munns R, Nishizawa NK (2013). Using membrane transporters to improve crops for sustainable food production. Nature..

[3] Blumwald E (2000). Sodium transport and salt tolerance in plants. Curr Opin Cell Biol.

[4] Volkov V (2015). Salinity tolerance in plants. Quantitative approach to ion transport starting from halophytes and stepping to genetic and protein engineering for manipulating ion fluxes. Front Plant Sci.

[5] Bassil E, Blumwald E (2014). The ins and outs of intracellular ion homeostasis: NHX-type cation/H^+^ transporters. Curr Opin Plant Biol.

[6] Shi H, Ishitani M, Kim C, Zhu JK (2000). The *Arabidopsis thaliana* salt tolerance gene *SOS1* encodes a putative Na^+^/H^+^ antiporter. Proc Natl Acad Sci U S A.

[7] El Mahi H, Perez-Hormaeche J, De Luca A, Villalta I, Espartero J, Gamez-Arjona F, Fernandez JL, Bundo M, Mendoza I, Mieulet D (2019). A critical role of sodium flux via the plasma membrane Na^+^/H^+^ exchanger SOS1 in the salt tolerance of rice. Plant Physiol.

[8] Olias R, Eljakaoui Z, Pardo JM, Belver A (2009). The Na^+^/H^+^ exchanger SOS1 controls extrusion and distribution of Na^+^ in tomato plants under salinity conditions. Plant Signal Behav.

[9] Zhu JK (2000). Genetic analysis of plant salt tolerance using *Arabidopsis*. Plant Physiol.

[10] Guo Y, Halfter U, Ishitani M, Zhu JK (2001). Molecular characterization of functional domains in the protein kinase SOS2 that is required for plant salt tolerance. Plant Cell.

[11] Kumar S, Kalita A, Srivastava R, Sahoo L (2017). Co-expression of *Arabidopsis* NHX1 and bar improves the tolerance to salinity, oxidative stress, and herbicide in transgenic mungbean. Front Plant Sci.

[12] Moghaieb RE, Sharaf AN, Soliman MH, El-Arabi NI, Momtaz OA (2014). An efficient and reproducible protocol for the production of salt tolerant transgenic wheat plants expressing the *Arabidopsis AtNHX1* gene. GM Crops Food.

[13] Zeng Y, Li Q, Wang H, Zhang J, Du J, Feng H, Blumwald E, Yu L, Xu G (2018). Two NHX-type transporters from *Helianthus tuberosus* improve the tolerance of rice to salinity and nutrient deficiency stress. Plant Biotechnol J.

[14] Qiu QS (2016). Plant endosomal NHX antiporters: activity and function. Plant Signal Behav.

[15] Bassil E, Ohto MA, Esumi T, Tajima H, Zhu Z, Cagnac O, Belmonte M, Peleg Z, Yamaguchi T, Blumwald E (2011). The *Arabidopsis* intracellular Na^+^/H^+^ antiporters NHX5 and NHX6 are endosome associated and necessary for plant growth and development. Plant Cell.

[16] Zhang L, Ma H, Chen T, Pen J, Yu S, Zhao X (2014). Morphological and physiological responses of cotton (*Gossypium hirsutum* L.) plants to salinity. PLoS One.

[17] Ashraf J, Zuo D, Wang Q, Malik W, Zhang Y, Abid MA, Cheng H, Yang Q, Song G (2018). Recent insights into cotton functional genomics: progress and future perspectives. Plant Biotechnol J.

[18] Cominelli E, Conti L, Tonelli C, Galbiati M (2013). Challenges and perspectives to improve crop drought and salinity tolerance. New Biotechnol.

[19] Mittler R, Blumwald E (2010). Genetic engineering for modern agriculture: challenges and perspectives. Annu Rev Plant Biol.

[20] Peng Z, He S, Gong W, Sun J, Pan Z, Xu F, Lu Y, Du X (2014). Comprehensive analysis of differentially expressed genes and transcriptional regulation induced by salt stress in two contrasting cotton genotypes. BMC Genomics.

[21] Long L, Yang W, Liao P, Guo Y, Kumar A, Gao W (2019). Transcriptome analysis reveals differentially expressed ERF transcription factors associated with salt response in cotton. Plant Sci.

[22] Xu F, Liu H, Xu Y, Zhao J, Guo Y, Long L, Gao W, Song C (2018). Heterogeneous expression of the cotton R2R3-MYB transcription factor GbMYB60 increases salt sensitivity in transgenic *Arabidopsis*. Plant Cell Tissue Organ Cult.

[23] Zhang T, Hu Y, Jiang W, Fang L, Guan X, Chen J, Zhang J, Saski CA, Scheffler BE, Stelly DM (2015). Sequencing of allotetraploid cotton (*Gossypium hirsutum* L. acc. TM-1) provides a resource for fiber improvement. Nat Biotechnol.

[24] Wang M, Tu L, Yuan D, Zhu D, Shen C, Li J, Liu F, Pei L, Wang P, Zhao G (2019). Reference genome sequences of two cultivated allotetraploid cottons, *Gossypium hirsutum* and *Gossypium barbadense*. Nat Genet.

[25] Gao W, Long L, Tian X, Xu F, Liu J, Singh PK, Botella JR, Song C (2017). Genome editing in cotton with the CRISPR/Cas9 system. Front Plant Sci.

[26] Long L, Guo D, Gao W, Yang W, Hou L, Ma X, Miao Y, Botella JR, Song C (2018). Optimization of CRISPR/Cas9 genome editing in cotton by improved sgRNA expression. Plant Methods.

[27] Gao W, Xu F, Long L, Li Y, Zhang J, Chong L, Botella JR, Song C. The gland localized CGP1 controls gland pigmentation and gossypol accumulation in cotton. Plant Biotechnol J. 2020. 10.1111/pbi.13323. 10.1111/pbi.13323 PMC7292540 31883409

[28] Chen X, Lu X, Shu N, Wang D, Wang S, Wang J, Guo L, Guo X, Fan W, Lin Z (2017). *GhSOS1*, a plasma membrane Na^+^/H^+^ antiporter gene from upland cotton, enhances salt tolerance in transgenic *Arabidopsis thaliana*. PLoS One.

[29] Mu C, Zhou L, Shan L, Li F, Li Z (2019). Phosphatase GhDsPTP3a interacts with annexin protein GhANN8b to reversely regulate salt tolerance in cotton (*Gossypium* spp.). New Phytol.

[30] Cao B, Long D, Zhang M, Liu C, Xiang Z, Zhao A (2016). Molecular characterization and expression analysis of the mulberry Na^+^/H^+^ exchanger gene family. Plant Physiol Biochem.

[31] Fukuda A, Nakamura A, Hara N, Toki S, Tanaka Y (2011). Molecular and functional analyses of rice NHX-type Na^+^/H^+^ antiporter genes. Planta..

[32] Hima Kumari P, Anil Kumar S, Ramesh K, Sudhakar Reddy P, Nagaraju M, Bhanu Prakash A, Shah T, Henderson A, Srivastava RK, Rajasheker G (2018). Genome-wide identification and analysis of *Arabidopsis* sodium proton antiporter (NHX) and human sodium proton exchanger (NHE) homologs in *Sorghum bicolor*. Genes..

[33] Sandhu D, Pudussery MV, Kaundal R, Suarez DL, Kaundal A, Sekhon RS (2018). Molecular characterization and expression analysis of the Na^+^/H^+^ exchanger gene family in *Medicago truncatula*. Funct Integr Genomics.

[34] Yokoi S, Quintero FJ, Cubero B, Ruiz MT, Bressan RA, Hasegawa PM, Pardo JM (2002). Differential expression and function of *Arabidopsis thaliana* NHX Na^+^/H^+^ antiporters in the salt stress response. Plant J.

[35] Sharma H, Taneja M, Upadhyay SK (2020). Identification, characterization and expression profiling of cation-proton antiporter superfamily in *Triticum aestivum* L. and functional analysis of TaNHX4-B. Genomics..

[36] Li F, Fan G, Wang K, Sun F, Yuan Y, Song G, Li Q, Ma Z, Lu C, Zou C (2014). Genome sequence of the cultivated cotton *Gossypium arboreum*. Nat Genet.

[37] Wang K, Wang Z, Li F, Ye W, Wang J, Song G, Yue Z, Cong L, Shang H, Zhu S (2012). The draft genome of a diploid cotton *Gossypium raimondii*. Nat Genet.

[38] Gao W, Xu F, Guo D, Zhao J, Liu J, Guo Y, Singh PK, Ma X, Long L, Botella JR (2018). Calcium-dependent protein kinases in cotton: insights into early plant responses to salt stress. BMC Plant Biol.

[39] Jauh GY, Fischer AM, Grimes HD, Ryan CA, Rogers JC (1998). delta-Tonoplast intrinsic protein defines unique plant vacuole functions. Proc Natl Acad Sci U S A.

[40] Munns R, Gilliham M (2015). Salinity tolerance of crops - what is the cost?. New Phytol.

[41] Qin YM, Zhu YX (2011). How cotton fibers elongate: a tale of linear cell-growth mode. Curr Opin Plant Biol.

[42] Schmutz J, Cannon SB, Schlueter J, Ma J, Mitros T, Nelson W, Hyten DL, Song Q, Thelen JJ, Cheng J (2010). Genome sequence of the palaeopolyploid soybean. Nature..

[43] Paterson AH, Wendel JF, Gundlach H, Guo H, Jenkins J, Jin D, Llewellyn D, Showmaker KC, Shu S, Udall J (2012). Repeated polyploidization of *Gossypium* genomes and the evolution of spinnable cotton fibres. Nature..

[44] Wang X, Guo H, Wang J, Lei T, Liu T, Wang Z, Li Y, Lee TH, Li J, Tang H (2016). Comparative genomic de-convolution of the cotton genome revealed a decaploid ancestor and widespread chromosomal fractionation. New Phytol.

[45] Sze H, Chanroj S (2018). Plant endomembrane dynamics: studies of K+/H+ antiporters provide insights on the effects of pH and ion homeostasis. Plant Physiol.

[46] Shang X, Cao Y, Ma L (2017). Alternative splicing in plant genes: a means of regulating the environmental fitness of plants. Int J Mol Sci.

[47] Laloum T, Martin G, Duque P (2018). Alternative splicing control of abiotic stress responses. Trends Plant Sci.

[48] Cooper TA (2005). Alternative splicing regulation impacts heart development. Cell..

[49] Yu J, Jung S, Cheng CH, Ficklin SP, Lee T, Zheng P, Jones D, Percy RG, Main D (2014). CottonGen: a genomics, genetics and breeding database for cotton research. Nucleic Acids Res.

[50] Krzywinski M, Schein J, Birol I, Connors J, Gascoyne R, Horsman D, Jones SJ, Marra MA (2009). Circos: an information aesthetic for comparative genomics. Genome Res.

[51] Sturn A, Quackenbush J, Trajanoski Z (2002). Genesis: cluster analysis of microarray data. Bioinformatics..

[52] Gao W, Long L, Xu L, Lindsey K, Zhang X, Zhu L (2016). Suppression of the homeobox gene *HDTF1* enhances resistance to *Verticillium dahliae* and *Botrytis cinerea* in cotton. J Integr Plant Biol.

[53] Long L, Xu F, Zhao J, Li B, Xu L, Gao W. GbMPK3 overexpression increases cotton sensitivity to *Verticillium dahliae* by regulating salicylic acid signaling. Plant Sci. 2020. 10.1016/j.plantsci.2019.110374. 10.1016/j.plantsci.2019.110374 32005380

[54] Bai L, Ma X, Zhang G, Song S, Zhou Y, Gao L, Miao Y, Song C (2014). A receptor-like kinase mediates ammonium homeostasis and is important for the polar growth of root hairs in *Arabidopsis*. Plant Cell.

[55] Ma X, Zhang X, Yang L, Tang M, Wang K, Wang L, Bai L, Song C (2019). Hydrogen peroxide plays an important role in PERK4-mediated abscisic acid-regulated root growth in *Arabidopsi*s. Funct Plant Biol.

[56] Long L, Zhao J, Xu F, Yang W, Liao P, Gao Y, Gao W, Song C (2018). Silencing of *GbANS* reduces cotton resistance to *Verticillium dahliae* through decreased ROS scavenging during the pathogen invasion process. Plant Cell Tissue Organ Cult.

[57] Rus A, Yokoi S, Sharkhuu A, Reddy M, Lee BH, Matsumoto TK, Koiwa H, Zhu JK, Bressan RA, Hasegawa PM (2001). AtHKT1 is a salt tolerance determinant that controls Na+ entry into plant roots. Proc Natl Acad Sci U S A.

