# Supplementary figures and images for: Identification of NHXs in Gossypium species and the positive role of GhNHX1 in salt tolerance
Source: BMC Plant Biol. 2020 Apr 8;20:147. doi: 10.1186/s12870-020-02345-z (PMC7140369; doi:10.1186/s12870-020-02345-z)

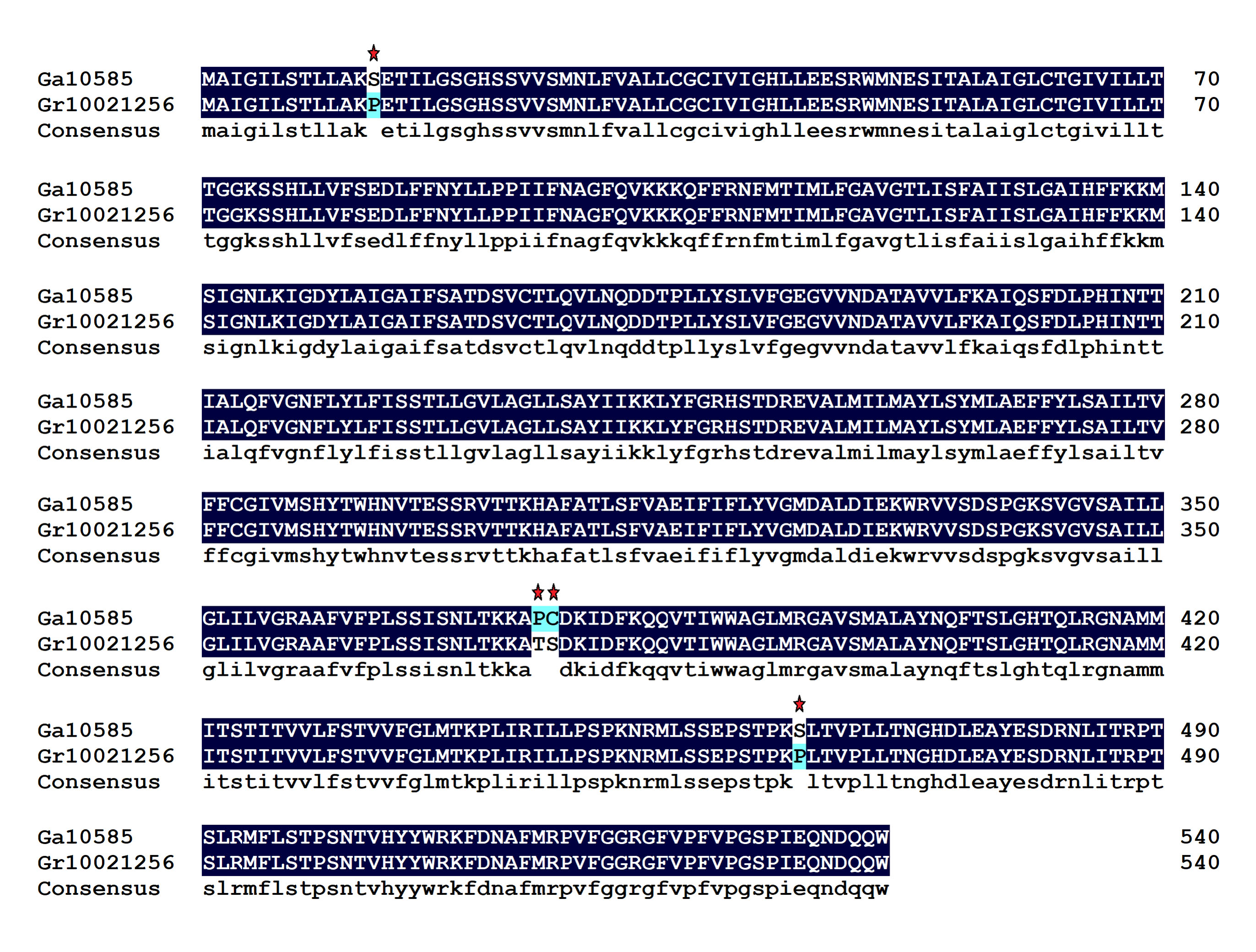

Supplement: Supplementary file 4 — Additional file 4: Figure S1. Sequence alignment of Ga10585 and Gr10021256. The alignments were performed using DNAMAN software. Identical amino acids are in dark blue background. The sequence differences are indicated by red asterisk. [file 12870_2020_2345_MOESM4_ESM.jpg]

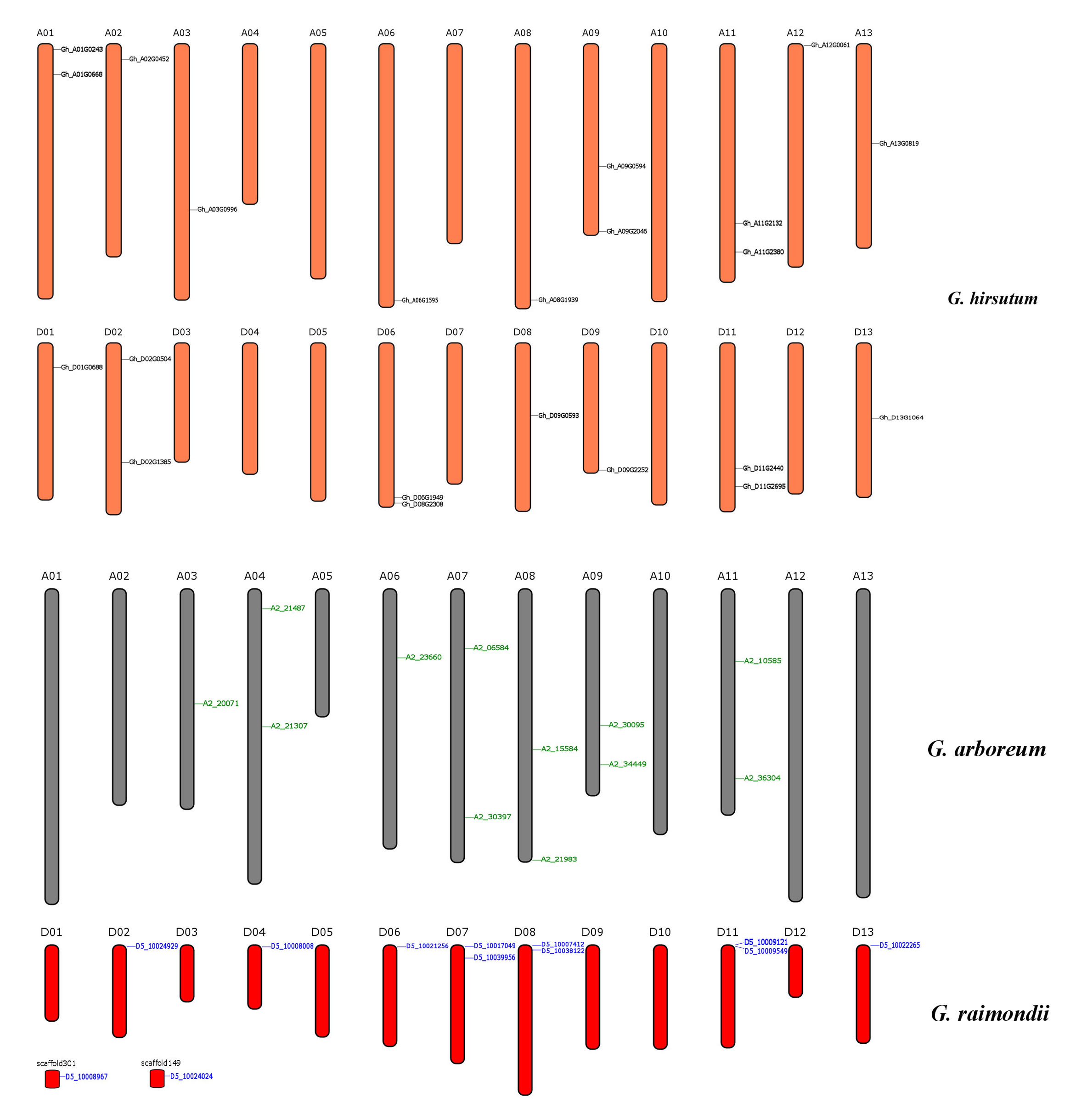

Supplement: Supplementary file 5 — Additional file 5: Figure S2. Chromosomal distribution of NHXs from G. hirsutum, G. arboreum, and G. raimondii. [file 12870_2020_2345_MOESM5_ESM.jpg]

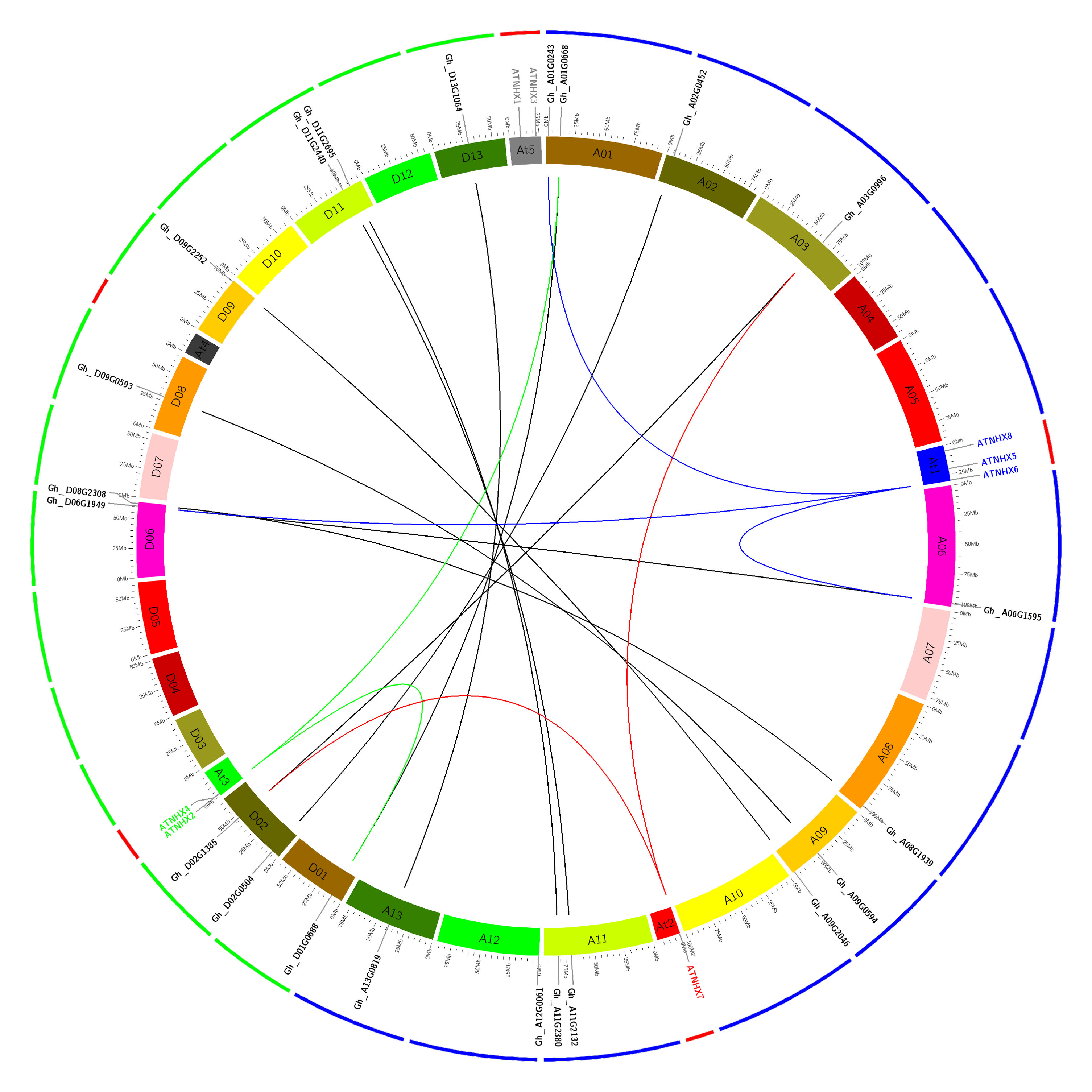

Supplement: Supplementary file 6 — Additional file 6: Figure S3. Genome-wide synteny analysis of NHXs from G. hirsutum and Arabidopsis. The approximate positions of NHXs in chromosomes are indicated by a short gray line on the Circos circle with different colors representing different chromosomes. [file 12870_2020_2345_MOESM6_ESM.jpg]

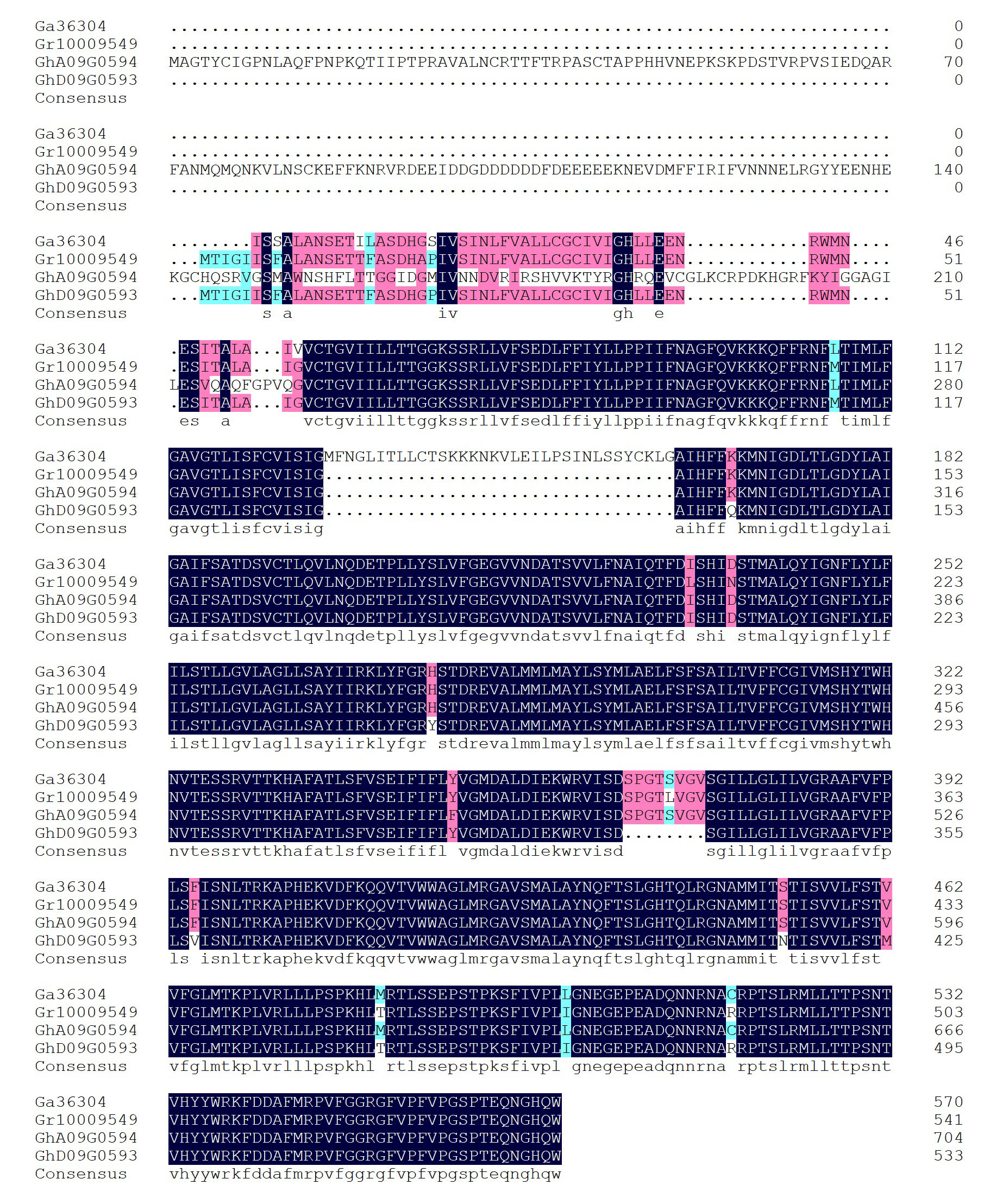

Supplement: Supplementary file 7 — Additional file 7: Figure S4. Sequence alignment of Ga36304, GhA09G0594, Gr10009549, and GhD09G0593. The alignments were performed using DNAMAN software. Identical amino acids are in dark blue background. [file 12870_2020_2345_MOESM7_ESM.jpg]
